# Influence of gastric endoscopic submucosal dissection on serum opsonic activity measured by chemiluminescence

**DOI:** 10.3164/jcbn.18-93

**Published:** 2019-02-06

**Authors:** Tetsu Arai, Daisuke Chinda, Tadashi Shimoyama, Kaori Sawada, Kazuki Akitaya, Kuniaki Miyazawa, Naoki Akimoto, Satoshi Sato, Shiro Hayamizu, Tetsuya Tatsuta, Hidezumi Kikuchi, Hiroto Hiraga, Manabu Sawaya, Hirotake Sakuraba, Tatsuya Mikami, Shigeyuki Nakaji, Shinsaku Fukuda

**Affiliations:** 1 Department of Gastroenterology and Hematology, Hirosaki University Graduate School of Medicine, 5 Zaifu-cho, Hirosaki, Aomori 036-8562, Japan; 2 Department of Community Medicine, Hirosaki University Graduate School of Medicine, 5 Zaifu-cho, Hirosaki, Aomori 036-8562, Japan; 3 Aomori General Health Examination Center, 2-19-12 Tsukuda, Aomori, Aomori 030-0962, Japan; 4 Department of Social Medicine, Hirosaki University Graduate School of Medicine, 5 Zaifu-cho, Hirosaki, Aomori 036-8562, Japan; 5 Division of Endoscopy, Hirosaki University Hospital, 52 Hon-cho, Hirosaki, Aomori 036-8563, Japan

**Keywords:** endoscopic submucosal dissection, serum opsonic activity, chemiluminescence, early gastric cancer

## Abstract

This study aimed to elucidate whether changes in serum opsonic activity measured by lucigenin-dependent chemiluminescence and luminol-dependent chemiluminescence are useful for estimating physical stress during the perioperative period of gastric endoscopic submucosal dissection. Serum opsonic activity in the peripheral blood of 87 patients was examined in the morning of the day of endoscopic submucosal dissection, the next day, and at 4 days after endoscopic submucosal dissection. Peak height and area under the curve for lucigenin-dependent chemiluminescence were 106.1 ± 22.7% and 102.0 ± 24.7% on the day of endoscopic submucosal dissection, which increased significantly to 113.6 ± 29.4% and 111.0 ± 29.1% on the next day (both *p*<0.01), and 112.4 ± 27.0% and 110.0 ± 28.1% at 4 days after endoscopic submucosal dissection (both *p*<0.01), respectively. In contrast, significant changes were not observed in peak height and area under the curve for luminol-dependent chemiluminescence during the perioperative period of endoscopic submucosal dissection. This difference suggests that serum opsonic activity during the perioperative period of gastric endoscopic submucosal dissection is associated with the production of substances with lower oxidizing potential. (The study of changes in neutrophil function and physical stress during the perioperative period of endoscopic operation: UMIN000034514)

## Introduction

Endoscopic submucosal dissection (ESD) is recognized as a safe and useful treatment for early gastric cancer.^(1–5)^ Several studies have measured physical stress associated with ESD; however, only few studies have assessed physical stress during the perioperative period of ESD using blood samples. Our previous studies, which measured the change in energy metabolism using an indirect calorimeter and Long’s method, revealed that the degree of invasion during the perioperative period of ESD was lower than that during surgery.^(6,7)^ Another study also showed that salivary amylase activity was increased by the physical stress associated with gastric ESD, including pain.^(8)^ Previously, the invasiveness of surgery had been evaluated by measuring the levels of catecholamine, cortisol, adrenocorticotropic hormone, blood sugar, blood insulin or interleukin-6.^(9–12)^ However, these factors have not been firmly established for the measurement of invasiveness of surgery because of varying results across studies.

Production of reactive oxygen species (ROS) by neutrophils has been shown to be related to physical stress.^(13–16)^ Phagocytosis by neutrophils followed by ROS production plays an important role in non-specific immunity. Adhesion of immunoglobulins and complements to foreign bodies is called ‘opsonization’ and activates phagocytosis and subsequent ROS production by neutrophils. In the field of sports medicine, serum opsonic activity (SOA) has been measured using chemiluminescence (CL) to evaluate the stress of exercises. CL has been widely used as a sensitive and accurate method for the assessment of the capacity of neutrophils to produce ROS.^(16–18)^ The species and amount of ROS can be measured using both lucigenin-dependent chemiluminescence (LgCL) and luminol-dependent chemiluminescence (LmCL).^(14–16,19)^ Lucigenin is well associated with the detection of superoxide anions (O_2_^•−^), whereas luminol relatively reflects the total production of ROS including hypochlorous acid (HOCl), which has higher oxidizing potential and is produced by myeloperoxidase (MPO). However, to date, the influence of gastric ESD on SOA has not been studied.

The aim of this study was to elucidate whether the changes in SOA measured by LgCL and LmCL are useful for estimating physical stress during the perioperative period of gastric ESD. Furthermore, we examined the change in serum levels of immunoglobulins and complements after gastric ESD.

## Materials and Methods

### Patients

**Fig. 1:**
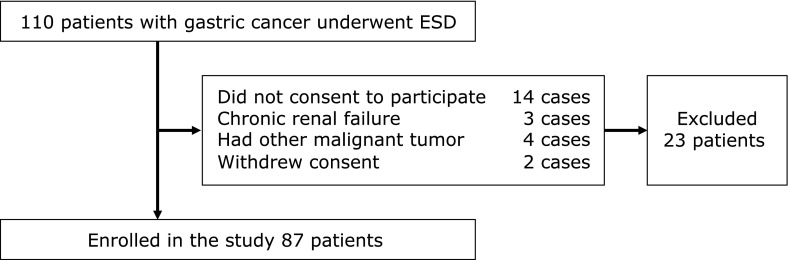
Flowchart of this study.

Patients who underwent ESD in Hirosaki University Hospital from January 2016 to February 2017 were prospectively entered into the study. During the study period, 110 consecutive patients were enrolled. We excluded patients who had a history of liver cirrhosis, were undergoing artificial dialysis, had other malignancies, and/or were using immunomodulatory drugs because SOA would be modulated under these conditions.^(20–23)^ Finally, 87 patients (mean age: 73.5 ± 7.6 years, 59 male patients) were studied (Fig. 1). ESD was performed using a conventional single channel endoscope (GIF-Q260J, GIF-H260 or H290; Olympus, Tokyo, Japan) with hood. The ESD procedure was performed using a water jet short needle-knife with a small ball tip (Flush Knife BT-S; DK2620J, Fujinon, Tokyo, Japan), a water jet hook knife (Hook Knife J; KD-625, Olympus, Tokyo, Japan), and a high frequency generator with an automatically controlled system, ICC200 or VIO300D (both supplied by ERBE, Tübingen, Germany). In all patients, we initially used pethidine hydrochloride (25–100 mg/patient), together with midazolam or diazepam. All ESD procedures were performed by board-certified endoscopists of the Japan Gastroenterological Endoscopy Society.

Blood samples were obtained in the morning on the day of ESD, the next day, and 4 days after ESD, under fasting over 12 h and non-smoking condition over 24 h, while resting in bed. Blood sampling was performed from the intermediate antebrachial vein using a vacuum blood collection tube. Serum samples were separated by centrifugation at 3,000 rpm for 10 min, after allowing the blood to clot for 30 min at room temperature. Serum samples were stored at –80°C until analysis. The number and differential counts of white blood cells (WBC) was analyzed by an XE-5000 (Sysmex, Kobe, Japan). The serum level of C-reactive protein (CRP) was measured by a JCA-BM6070 (EOL Ltd., Tokyo, Japan). The serum levels of immunoglobulins (lgG, IgM, and IgA), complements (C3 and C4), and 50% hemolytic complement activity (CH50) were also measured by turbidimetric immunoassay.

### LgCL and LmCL assay

As previously described,^(24–27)^ SOA in the peripheral blood of the patients was examined by measuring neutrophil ROS production using LgCL and LmCL. Neutrophils were isolated from the peripheral blood of a healthy adult man under non-smoking conditions over 12 h using Mono-Poly Resolving Medium (Dainippon Pharmaceutical, Tokyo, Japan) just before the CL assay. Neutrophil suspensions were prepared by adjusting neutrophil counts to 3.0 × 10^3^ cells/µl through dilution with Hanks’ Balanced Salt Solution (HBSS).^(26–28)^ Zymosan obtained from *Saccharomyces cerevisiae*, a well-known activator of the alternative pathway of the complements system, was employed for opsonized particles. In this study, opsonized zymosan (OZ) was prepared by the following method. Zymosan A (Sigma, St. Louis, MO) was suspended in HBSS at a concentration of 5 mg/ml followed by sonication. Two-hundred fifty microliters of serum samples were added to suspended zymosan (5 mg/ml) and incubated at 37°C for 30 min. Two chemiluminigenic probes, lucigenin and luminol, were used for the detection of ROS. Lucigenin was prepared by dissolving bus-*N*-methylacridinium nitrate (Sigma) in HBSS to give a final concentration of 0.5 mM (pH 7.4). Luminol was prepared by dissolving 5-amino-2,3-dihydro-1,4-phatalazinedione (Sigma) initially in 0.1 M NaOH to give a clear solution and then adjusted using HCl and HBSS to give a final concentration of 0.5 mM (pH 7.4). CL responses of each sample were studied in a 96-well microplate (well capacity 400 µl; Greiner Japan, Tokyo, Japan) simultaneously. To each well of a microplate, 50-µl neutrophil suspension, 50-µl OZ, 50-µl lucigenin or luminol solution, and 100-µl HBSS were added. CL was measured continuously at 37°C using an Auto Luminescence Analyzer, Alfa system (Tokken, Funabashi, Japan).^(29)^ CL assays were evaluated using the maximum light emission (peak height; PH) and area under the emission curve (area under the curve; AUC). In each CL assay, ROS production was also measured using a stored serum from a healthy male volunteer to serve as a standard value. Results of CL assay were expressed as the percentage to the values obtained from the standard serum.

### Ethics

This study was approved by the Hirosaki University Ethics Committee. Prior to the admission or on the day before ESD procedure, the details of the investigation procedure and the research objective were explained to the participants, and written informed consent was obtained from all participants who were willing to collaborate.

### Statistical analysis

The results of hematological and biochemical assays were expressed as means ± SD, and were analyzed using the paired Student’s *t* test. PH and AUC of CL assays were analyzed using the Wilcoxon-signed rank test comparing perioperative alterations with preoperative values. All *p* values were two-tailed, and *p*<0.05 was considered statistically significant.

## Results

### Patient characteristics

Table 1 shows the characteristics of the patients. Target sample size was 78 when the significance level was set at 5% and the power at 80%. The final sample size of this study was 87, and the power of this analysis was calculated as 84.5%.

The resection area was computed by approximation as an ellipse with the length and breadth of the resection specimen. In the 27 patients with multiple lesions, the total resection area of all lesions was computed. Postoperative complications were as follows: perforation in 1 patient (1.1%), postoperative bleeding in 4 (4.6%), and fever >38°C in 7 (8.0%). All patients were treated successfully, and none required additional surgical treatment.

### Serial changes in peripheral leukocytes, neutrophil count and CRP

WBC and the number of neutrophils increased significantly on the next day and at 4 days after ESD compared to those on the day of ESD (*p*<0.001, Table 2). The increase in WBC and neutrophils was smaller at 4 days after ESD than on the next day of ESD. CRP increased significantly on the next day of ESD (*p*<0.001), and further increase was observed at 4 days after ESD (*p*<0.001).

### Perioperative changes in the serum

Table 3 shows the changes in the serum levels of immunoglobulins and complements. The IgG level decreased significantly on the next day and at 4 days after ESD compared with that on the day of ESD (*p*<0.001). However, no significant changes were observed in the IgA level. The IgM level decreased significantly on the next day (*p*<0.01), but recovered at 4 days after ESD.

The C3 and C4 levels increased significantly on the next day of ESD (*p*<0.05, *p*<0.001, respectively) and at 4 days after ESD (*p*<0.001, respectively). The CH50 level increased significantly at 4 days after ESD (*p*<0.001), although there were no differences between the day of ESD and the next day of ESD.

### Perioperative changes in SOA

**Fig. 2:**
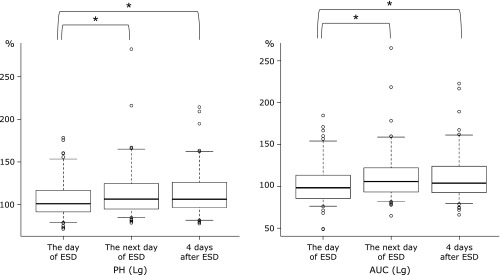
Changes in PH and AUC of LgCL. Results on the next day of ESD and at 4 days after ESD are compared with those on the day of ESD. Results are expressed by percentages of the values obtained from standard serum as 100%. PH, peak height; AUC, area under the curve; LgCL, lucigenin-dependent chemiluminescence; ESD, endoscopic submucosal dissection. *p<0.01.

**Fig. 3:**
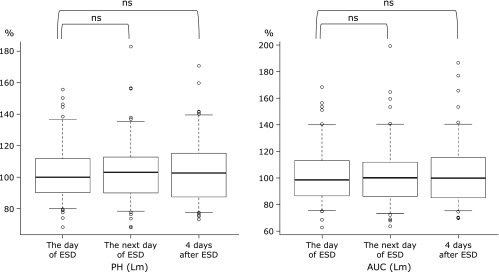
Changes in PH and AUC of LmCL. Results on the next day of ESD and at 4 days after ESD are compared with those on the day of ESD. Results are expressed by percentages of the values obtained from standard serum as 100%. PH, peak height; AUC, area under the curve; LmCL, luminol-dependent chemiluminescence; ESD, endoscopic submucosal dissection.

Figure 2 shows the PH and AUC of LgCL. PH of LgCL was 106.1 ± 22.7% on the day of ESD and increased to 113.6 ± 29.4% on the next day (*p*<0.01), and to 112.4 ± 27.0% at 4 days after ESD (*p*<0.01). The AUC of LgCL was 102.0 ± 24.7% on the day of ESD, and a significant increase was observed on the next day (111.0 ± 29.1%; *p*<0.01) and at 4 days after ESD (110.0 ± 28.1%; *p*<0.01). Both PH and AUC tended to decrease at 4 days after ESD compared to those on the next day of ESD. However, as shown in Fig. 3, significant changes were not observed in PH and AUC of LmCL during the perioperative period. The PH and AUC of Lm CL were 98.9 ± 20.6% and 100.7 ± 17.8% on the day of ESD, 103.8 ± 19.3% and 102.3 ± 22.5% on the next day, and 102.3 ± 22.3% and 103.7 ± 19.1% at 4 days after ESD, respectively.

We also examined the influence of total resection area, operation time, and histological type on the change of SOA (Table 4). Total resection area and operation time was divided into two groups according to their mean values (mean total resection area: 12.0 cm^2^, mean operation time: 123.8 min). The histological type of the tumor was also divided into two groups: tub1 or other types. In all groups, AUC measured by LgCL increased both on the next day and at 4 days after ESD compared with that on the day of ESD. However, AUC was not significantly different in all groups.

## Discussion

In this study, SOA to stimulate neutrophil ROS production was increased during the perioperative period of ESD for gastric cancer. SOA was highest on the next day of ESD and continued to increase even at 4 days after ESD. Therefore, the increase in SOA after ESD might not only be caused by the physical stress of the procedure but also by gastric mucosal inflammation, including post-ESD ulceration.

In previous studies, changes in SOA were assessed using LgCL and LmCL. Neutrophils produce O_2_^•−^ mediated by NADPH oxidase activated by phagocytosis, and O_2_^•−^ is rapidly converted to hydrogen peroxide (H_2_O_2_) either spontaneously or by superoxide dismutase. On the other hand, neutrophil azurophilic granules contain large quantities of MPO, which is discharged by the process of degranulation and reacts with H_2_O_2_ and Cl^−^ to produce HOCl. These ROS produced by neutrophils have different toxicities. HOCl has a significantly higher oxidizing potential than its precursors O_2_^•−^ and H_2_O_2_.^(15,18)^ LgCL reflects the production of O_2_^•−^, whereas LmCL is recognized to reflect total ROS production including HOCl. SOA measured by LgCL was reported to be increased by intense exercise and training including rugby sevens matches,^(30)^ long-term judo training,^(31)^ and intense sumo training.^(28)^ With respect to SOA measured by LmCL, no significant changes were observed in the perioperative period of spinal surgery, which is consistent with the results observed in our study.^(32)^ From the results obtained by LgCL and LmCL in this study, the change in SOA during the perioperative period of gastric ESD may enhance production of substances with lower oxidizing potential, such as O_2_^•−^, rather than those with higher oxidizing potential.

In the present study, the number of neutrophils increased significantly on the next day of ESD and at 4 days after ESD. The increase in the number of circulating neutrophils in parallel to physical stress after the surgical operation has been well demonstrated.^(32–36)^ These studies showed 2.64- to 4.48-fold increase in peripheral neutrophil counts on the day after gastrectomy.^(35,37,38)^ On the other hand, the present study showed a 2.41-fold increase in peripheral neutrophil counts after gastric ESD. Therefore, the increase in inflammatory cytokines to accelerate neutrophil migration induced by the physical stress of ESD and gastric mucosal inflammation would be lower than that induced by gastric surgery. Immunoglobulins and complements play an important role in SOA. In this study, during the perioperative period of gastric ESD, serum levels of IgG, IgA, and IgM tended to decrease, whereas those of C3, C4, and CH 50 increased significantly. With respect to the changes in neutrophil counts, immunoglobulins seemed to be consumed by the inflammatory response, whereas complements were increased due to nonspecific immunity. Our previous study on metabolic changes in patients during the ESD perioperative period also indicated that the energy requirements increased slightly on the next day of gastric ESD.^(6)^ However, in the present study, total resection area, operation time, and tumor histological type were all not associated with the change in SOA. Therefore, in terms of the physical stress of ESD, the inflammatory response after the procedure could be more relevant than procedural characteristics itself as the size of the resection area and operation time.

This study has some limitations. First, this study was performed at a single institute. However, our ESD procedure was performed according to the standard methods approved by the health insurance system of Japan. Therefore, it is likely that similar results would be obtained even when multicenter studies are performed. Second, no control group was included in this study to assess the physical stress associated with surgery. However, it is difficult to perform surgery for small gastric cancers at an early stage. Finally, the physical stress assessed in this study cannot be entirely attributed to physical stress due to the ESD procedure. During the perioperative period, other factors such as fasting and sedation would also be associated with the observed physical stress.

In conclusion, SOA measured by CL can be used to estimate the physical stress of ESD safely and easily. Therefore, this method could be an option that allows the study of physical stress of new techniques with lower invasiveness in numerous clinical fields. The difference between LgCL and LmCL suggests that SOA during the perioperative period of gastric ESD is associated with the production of substances with lower oxidizing potential. The results support the recognition that ESD a less invasive procedure.

## Author Contributions

TA, DC and TS wrote the manuscript and interpreted the data. TA, KS, KA, KM, NA, SS and SH performed data management and analysis. DC conceived the study design. TT, HK, HH, MS, HS, TM, SN, and SF played a role in reviewing and revising the manuscript. All authors approved the final draft of the manuscript.

## Figures and Tables

**Table 1:** Patient characteristics

Parameter	
Total patients	87
Age (years)^a^	73.5 ± 7.6 (53–86)
Sex	
Male:Female	59:28
Patients with multiple lesions^b^	34 (39%)
	×2: 28, ×3: 6
Histopathological diagnosis of the total 127 lesions^b^	
Indication lesion	90 (70.0%)
Extended indication lesion	27 (21.2%)
No-indication lesion	10 (0.8%)
Total resection area (cm^2^)^a^	12.0 ± 7.6 (2.7–41.2)
Operation time (min)^a^	123.8 ± 54.2 (42–249)
Complications^b^	
Bleeding	3 (4.6%)
Perforation	1 (1.1%)
Fever (>38°C)	7 (8.0%)

^a^Data are presented as mean ± SD (range). ^b^Data are presented as the number of positive case (percentage).

**Table 2:** Changes in WBC, neutrophils and CRP

	The day of ESD	The next day of ESD	4 days after ESD
WBC (/µl)	5,184 ± 1,325	8,940 ± 2,285*	6,196 ± 1,407*
Neutrophils (/µl)	2,797 ± 99	6,735 ± 145*	3,921 ± 122*
CRP (mg/dl)	0.202 ± 0.617	1.102 ± 1.268*	2.806 ± 2.615*

Data are presented as mean ± SD. *p<0.001, vs the day of ESD. WBC, white blood cells; CRP, C-reactive protein; ESD, endoscopic submucosal dissection.

**Table 3:** Changes in the levels of immunoglobulin and complements

	The day of ESD	The next day of ESD	4 days after ESD
IgG (mg/dl)	1,214.5 ± 319.1	1,168.3 ± 300.6***	1,160.5 ± 290.5***
IgA (mg/dl)	258.6 ± 123.2	255.6 ± 130.8	255.4 ± 117.8
IgM (mg/dl)	70.5 ± 31.9	68.7 ± 31.5**	69.3 ± 31.7
C3 (mg/dl)	101.2 ± 21.8	103.2 ± 22.1*	122.2 ± 26.2***
C4 (mg/dl)	25.6 ± 7.1	27.1 ± 7.6***	35.1 ± 9.5***
CH50 (U/ml)	55.6 ± 16.3	56.5 ± 11.8	67.1 ± 13.7***

Data are presented as mean ± SD. *p<0.05, **p<0.01, ***p<0.001, vs the day of ESD. ESD, endoscopic submucosal dissection.

**Table 4:** The degree of SOA affected by total resection area, operation time and histopathology

		AUC measured by LgCL					
	*n*	The day of ESD	*p*	The next day of ESD	*p*	4 days after ESD	*p*
Resection area							
<12.0 cm^2^	56	103.1 ± 25.8	0.94	111.8 ± 32.4	0.73	107.9 ± 28.9	0.35
≥12.0 cm^2^	31	102.6 ± 23.0		109.5 ± 22.2		113.8 ± 26.5	
Operation time							
<123.8 min	47	103.1 ± 28.4	0.94	107.9 ± 24.1	0.28	107.5 ± 28.0	0.37
≥123.8 min	40	102.7 ± 19.8		114.6 ± 33.9		112.9 ± 28.3	
Histological type							
tub1	62	105.0 ± 26.5	0.21	113.0 ± 23.6	0.31	112.0 ± 30.3	0.29
tub2/por/sig	25	97.6 ± 18.8		106.0 ± 39.3		104.9 ± 21.3	

Data are presented as mean ± SD. AUC, area under the curve; ESD, endoscopic submucosal dissection; LgCL, lucigenin-dependent chemiluminescence; tub1, well differentiated type tubular adenocarcinoma; tub2 moderately differentiated type tubular adenocarcinoma; por/sig, poorly differentiated adenocarcinoma and signet ring cell carcinoma.

## Abbreviations

AUC: area under the curve
CH50: 50% hemolytic complement activity
CL: chemiluminescence
CRP: C-reactive protein
ESD: endoscopic submucosal dissection
HBSS: Hanks’ Balanced Salt Solution
HOCl: hypochlorous acid
H_2_O_2_: hydrogen peroxide
LgCL: lucigenin-dependent chemiluminescence
LmCL: luminol-dependent chemiluminescence
MPO: myeloperoxidase
OZ: opsonized zymosan
O_2_^•−^: superoxide anions
PH: peak height
ROS: reactive oxygen species
SD: standard deviation
SOA: serum opsonic activity
WBC: white blood cells
